# Extensive skull ossification after decompressive craniectomy in an elderly patient: A case report and literature review

**DOI:** 10.1097/MD.0000000000029015

**Published:** 2022-03-18

**Authors:** Huanhuan Yang, Man Liang, Lijian Su

**Affiliations:** *Department of Forensic Medicine, Tongji Medical College, Huazhong University of Science and Technology, Wuhan, Hubei, China.*

**Keywords:** case report, decompressive craniectomy, elderly patient, extensive skull ossification, spontaneous bone regeneration, traumatic brain injury

## Abstract

**Rationale:**

After severe traumatic brain injury, patients often present with signs of increased intracranial hypertension and partially require decompressive craniectomies. Artificial materials are usually required to repair skull defects and spontaneous skull ossification is rarely observed in adults.

**Patient concerns:**

This study reported a 64-year-old man was admitted to the hospital with a coma due to a traffic accident.

**Diagnosis:**

Emergency computed tomography (CT) examination upon admission showed a left temporo-occipital epidural hematoma with a cerebral hernia and skull fracture.

**Interventions:**

The patient underwent urgent craniotomy for hematoma removal and decompression under general anesthesia. The patient was discharged after 1 month of treatment.

**Outcomes:**

The patient returned to the hospital for skull repair 145 days after the craniotomy. Pre-operative CT showed island skull regeneration in the skull defect area; therefore, skull repair was postponed after clinical evaluation. Regular follow-up is required. Twenty-three months after surgery, head CT showed that the new skull had completely covered the defect area.

**Lesson:**

We collected other 11 similar cases of spontaneous human skull regeneration in a literature search to analyze the possible factors impacting skull regeneration. The analysis of the cases indicated that maintaining the integrity of the periosteum, dura, and blood vessels during craniotomy may play an important role in skull regeneration. Skull regeneration predominantly occurs in young patients with rapid growth and development; therefore, an appropriate postponement of the cranioplasty time under close monitoring could be considered for young patients with skull defects.

## 1. Introduction

Traumatic brain injury (TBI) is usually accompanied by various secondary brain injuries, among which intracranial hypertension is the most common cause of death and disability. Approximately 10% to 15% of patients with increased intracranial pressure have no response to mannitol, drugs, and auxiliary hyperventilation treatment^[1,2]^ and require decompressive craniectomy (DC) therapy to reduce the risk.^[3]^ Most patients require cranioplasty therapy to restore the normal structural and physiological state of the brain 3 to 6 months after DC. Studies have shown that the human skull still maintains the ability of spontaneous ossification before the age of 2 and almost loses after 6 years old.^[4-7]^ This study reported the case of an elderly male patient whose skull defect disappeared spontaneously after DC. Meanwhile, we collected other 11 similar cases of spontaneous skull regeneration from a literature search to explore the possible factors that affect skull regeneration.

## 2. Case report

A 64-year-old man was admitted to the hospital because of a severe traffic accident with a 6 × 5 cm scalp hematoma at the top of the left temporal lobe. Upon admission, the patient developed delirium. His Glasgow Coma Score was 6 points (E1V1M4), bilateral pupil diameter was approximately 3.0 mm, light reflection (+), bilateral Babinski sign (-), and muscle strength of the limbs could not be checked because of non-cooperation. His vital signs were relatively stable, with a heart rate of 87 beats/min, blood pressure of 185/110 mmHg, and respiratory rate of 20 breaths/min. Emergency computed tomography (CT) examination upon admission showed a large extradural hematoma in the left parietal-occipital region complicated by a cerebral hernia and multiple fractures of the skull and ribs (Fig. 1A). No other major injuries were noted.

**Figure:**
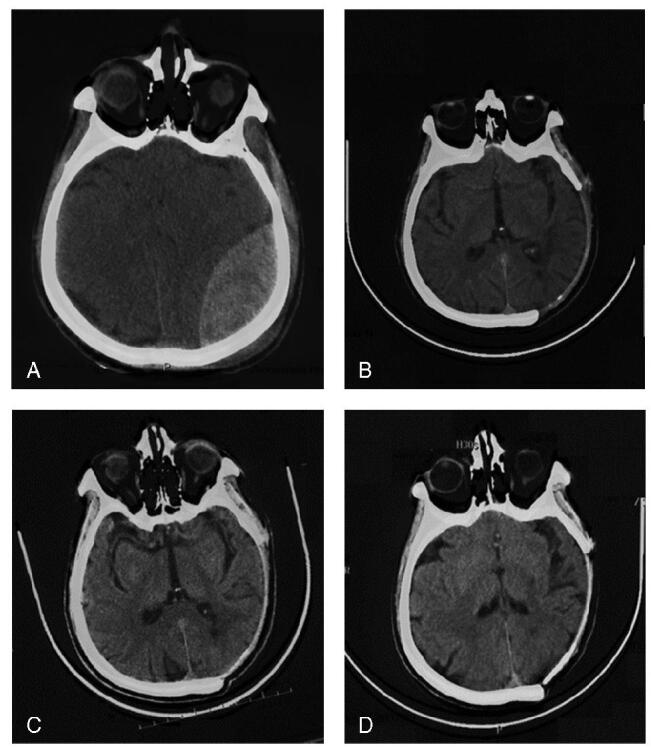
**Figure 1.** Heterotopic ossification (HO) progression. (A) CT on the day of injury showed a large extradural hematoma in the left tempo-parietal-occipital region; (B) CT at 145 days after DC showed the skull “island” regeneration in the defect area; (C) CT at 171 days after DC showed that the “island” skull regeneration expanded and fused to form a flake regeneration area; (D) CT at 23 months after DC showed the regenerated skull completely covers the defect area. CT = computed tomography, DC = decompressive craniectomy.

The patient underwent urgent craniotomy for hematoma removal and decompression under general anesthesia for approximately 20 minutes after admission. During the operation, a 15 cm horseshoe incision was made at the top of the left temporo-occipital lobe. The bone flap was separated layer-by-layer, and craniotomy was performed to form an 8 × 10 cm bone window. After craniotomy, a sizable extradural hematoma with a volume of approximately 40 mL was observed. No subdural hemorrhage was observed. The dura was unopened. The bone flap was removed, and 2 layers of the scalp were sealed: the inner layer consisted of the cranial capsule, dura mater, and gelatin sponge, and was sealed with discontinuous absorbable sutures; the outer layer consisted of skin, subcutaneous, and gelatin sponge, and was sealed with interrupted nonabsorbable sutures.

The patient recovered in the intensive care unit after surgery for 1 week and was transferred to the Department of Neurosurgery. The patient received prophylactic treatment for infection, gastrointestinal injury, and other supportive therapies. A CT scan at 3 days postoperation demonstrated no hematoma or active bleeding at the craniectomy site. The patient was discharged from the hospital after 1 month. His Glasgow coma score at discharge was 15 points without headache, dizziness, or any other lingering mental symptoms.

The patient returned to the hospital for a scheduled skull repair 145 days postoperatively. Pre-operative CT showed skull regeneration in the defect area, which was distributed as islands (Fig. 1B). Therefore, skull repair was postponed after clinical evaluation, and the patient was advised to receive monthly CT scans to monitor the regeneration of the skull. The head CT at 171 days postoperation showed that the “island” skull regeneration expanded and fused to form a flake regeneration area and nearly covered the entire defect area with varying thicknesses (Fig. 1C). Twenty-three months after the operation, the skull defect had completely disappeared (Fig. 1D). Physical examination showed that the left skull was hard but significantly sunk compared with the contralateral side (Fig. 2A, B).

**Figure:**
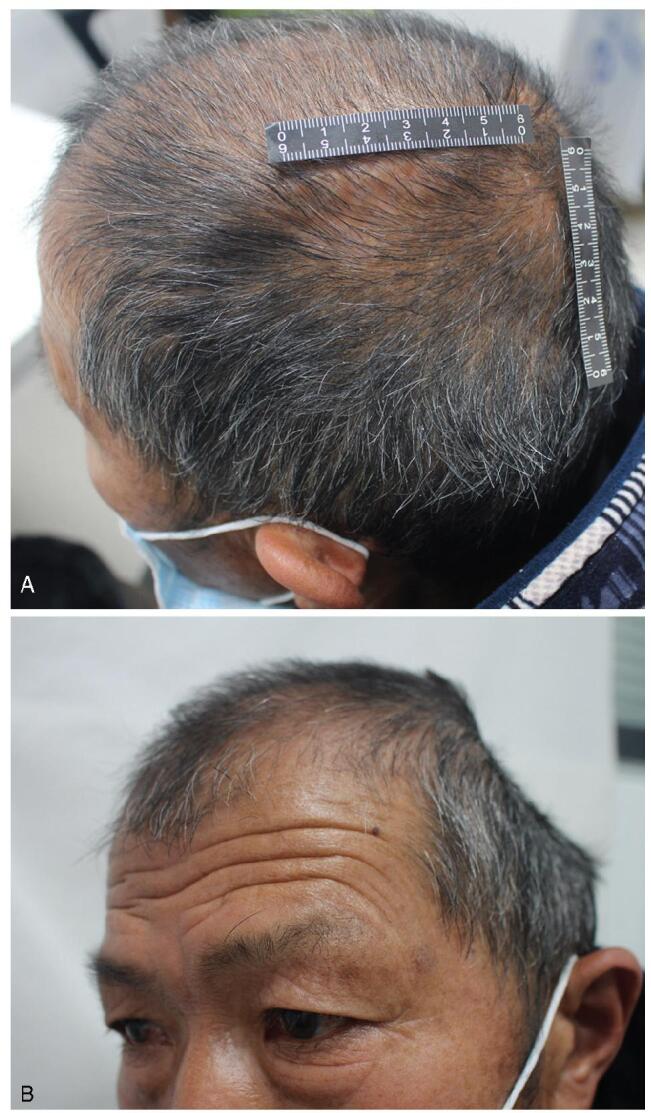
**Figure 2.** Head appearance at 23 months after DC. (A) Rear orthostatic photograph; (B) Lateral photograph. DC = decompressive craniectomy.

## 3. Literature review

### 3.1. Methods

We performed a literature search and review of extensive skull ossification in patients with severe TBI to identify whether there is a common pattern of skull regeneration.

A search of articles published from June 1991 through October 14, 2021, involving skull ossification was conducted via title and abstract search in the Web of Science, PubMed, and CNKI (a local database) databases. The search terms used were as follows: heterotopic ossification (HO), spontaneous bone regeneration, osteogenesis, decompressive craniectomy, and traumatic brain injury. Articles with clearly recorded information on patient sex, age, injury location, operation methods, skull defect, and time of skull regeneration were included and are presented in Table 1.

**Table 1:** General information of patients with spontaneous skull regeneration after TBI.

**Study**	**Gender**	**Age**	**Location of injuries**	**X-ray/CT report**	**Cranial defect area**	**Time of operation**	**Surgery procedure**	**Time of skull regeneration**
Greenwald et al, 2000^[6]^	Male	6	Bilateral frontal lobe	Diffuse subarachnoid hemorrhage; slit left epidural hematoma; signs of diffuse axonal injury	/	/	Bilateral c-shaped dural opening on the midline; Artificial dura was placed to complete a dura plasty; Pericranial flap placed over the dural layer.	On postoperative day (POD) 21, no ossification signs were observed; On POD 110, CT showed an abnormal hyperdense layer over both frontal lobes.
Figueroa-Sanchez et al, 2019^[4]^	Female	7	Frontotemporal lobe	Frontal fracture; open complex skull temporal fracture with dura mater lacerations	12 cm	/	Decompressive craniectomy was performed and loose bone fragments were removed; collagen dural substitute was used to complete a dura plasty.	Ten months postsurgery, well-defined “islands” of spontaneous bone regeneration in the craniectomy area were observed; At both 16- and 26-months postcraniectomy, incremental bone regeneration from the previously defined islands almost completely covered the calvarian defect.
Mathew and Chacko, 2008^[5]^	Female	12	Medulloblastoma^*^	/	/	/	Midline posterior fossa Craniectomy and radical excision of a vermian medulloblastoma.	Two years after craniectomy, the defect was almost completely covered by the new bone.
Li, 1998^[8]^	Male	17	Right parietal lobe	Dented comminuted fracture of the top of the right cranium	6 × 3.5 cm	On the day of injury	Conventional decompressive craniectomy.	Seven months after surgery, the skull regeneration was found to cover the defect area; One year after surgery, the edge of bone defect was palpable in physical examination. Six years after surgery, the regenerated bone plate was thickened and the margin of bone window almost disappeared.
Gao et al, 1991^[9]^	Male	17	Right frontal temporal lobe	/	8 × 10 cm	On the day of injury	Horseshoe-shaped craniotomy; the intact periosteum was preserved when the bone flap was removed and then sutured intermittently with the remaining periosteum along the wound.	On POD 194, arc-shaped eggshell change was revealed at the defect area; On POD 233, the skull defect was completely covered with regenerated bone with a thickness of 3 mm.
Hoover and Mahmood, 2001^[10]^	Male	17	Bilateral frontal lobe	Bilateral frontal lobe penetrating injury	/	On the day of injury	Bicoronal craniectomy and hematoma evacuation were performed; vascularized pericranial flap was used for duraplasty.	Five months after craniectomy, a large island of bone was encountered within the skull defect area.
Gao et al, 1991^[9]^	Male	18	Right frontotemporal lobe	Right frontotemporal comminuted fracture with intracerebral hematoma penetrating into cerebral ventricles and subdural hematoma	7 × 10 cm	On the day of injury	Craniotomy with cranial hematoma removal; the intact periosteum was preserved when the bone flap was removed and then sutured intermittently with the remaining periosteum along the wound.	On POD 264, intermittent bone regeneration was observed at the defect area; On POD 361, the defect was completely covered with the regenerated bone with thickness 2.5 mm to 5 mm.
Vega and Hutchins, 2017^[11]^	Male	18	Right frontal lobe	Right frontal contusions with diffuse axonal injury	/	Right side: 10 days postinjury; Left side: the exact time of operation is unknown	Decompressive craniectomy was conducted on both sides; the dura was cut in stellate fashion and a dura substitute was used for dura formation.	On POD 33 days, early calcification appeared on the right side, but not on the left; On POD 107, ossification began to appear on the left side; On 119 days postinjury, thick pieces of bone formed along the right ridge and thin sheets of bone formed along the dura mater itself on the left. On POD 160, bilateral calcification continued increasing.
Tran et al, 2021^[12]^	Female	20	Right temporal and parietal lobes	Extradural hematoma in the right tempoparietal-occipital region	10 × 10 cm	On the day of injury	Extradural hematoma evacuation with craniectomy was performed; the dura was unopen; the scalp was closed in 2-layer fashion: the inner layer (pericranium to the subcutaneous) was closed using interrupted absorbable sutures; the outer layer (skin and the subcutaneous) was closed using interrupted non-absorbable sutures.	At 7 weeks postoperation, thin bone ossification was found in the craniectomy region; At 23 weeks postoperation, CT scan revealed spontaneous cranial bone regeneration which nearly cover the whole craniectomy site.
Gao et al, 1991^[9]^	Male	24	Bilateral frontotemporal parietal lobes	Bilateral frontotemporal parietal fracture with anterior fossa base fracture	Right side: 6 × 11 cm Left side: 4 × 6 cm	On the day of injury	Right side: craniotomy was performed; the periosteum was not able to be repaired due to severely crushed skull and broken periosteum; Left side: decompressive craniotomy was performed; the intact periosteum was preserved when the bone flap was removed and then sutured intermittently with the remaining periosteum along the wound.	On POD 455, skull regeneration was observed at the left defect area with a thickness of 1 mm, while there was no change on the right side. On POD 547, there was no spontaneous cranial regeneration observed in the right defect area.
González-Bonet, 2021^[13]^	Female	29	Right frontal lobe	Acute right subdural hematoma associated with a brain contusion and a midline shift beyond 1 cm; A skull base fracture	/	On the day of injury	Decompressive craniectomy was performed with hematoma removal; collagen matrix was used as a patch between dura mater edges.	At the second year of follow up, CT scan showed a spontaneous bone formation in the large craniectomy defect.
CT = computed tomography, POD = postoperative day, TBI=traumatic brain injury.								

### 3.2. Results

Eleven studies on spontaneous skull regeneration after craniectomy were included. Among the patients, 8 were male (66.7%), and the mean age was 17.5 years old (range, 6-29years). Craniotomy was performed for craniocerebral trauma in 10 patients and medulloblastoma in 1 patient. Among the 10 patients with TBI, 7 patients underwent surgery on the day of injury, 1 patient underwent surgery 10 days postinjury, and 2 patients had an unknown time of operation. For the surgical methods, routine tension reduction suture was performed in 3 patients, and repair of the periosteum and dura mater using artificial material was performed in 7 patients. Among the 11 patients, 2 underwent midline skull resection, 3 underwent bilateral craniectomy, and the remaining 6 received unilateral craniectomy. In patients with regular follow-up, the earliest signs of skull regeneration were observed at 33 days after surgery, while the majority were at 0.5 to 1 year after surgery. Almost all patients had complete bone regeneration 2 years after surgery.

## 4. Discussion

Patients with severe TBI often present signs of intracranial hypertension and require partial DC treatment. Self-regeneration is rarely observed for skull defects larger than 4 cm in patients older than 6 years, and manual repair is usually required in these patients.

### 4.1. Possible mechanisms and impact factors in spontaneous skull regeneration

Three possible factors could contribute to spontaneous bone formation after large calvarial defects: pericranium/periosteum, diploe, and dura mater^[12,13]^; skull fracture can stimulate latent osteoblasts within the periosteum and on both sides of the defect,^[14]^ thus participating in the subsequent process of bone formation. Dura mater and pericranium/periosteum can enhance the effect of osteogenesis through contact with the graft.^[15]^ However, these hypotheses are mostly based on animal experiments, and there is a lack of relevant research on humans.

In the 12 cases in this study, all trauma patients ensured the integrity of the dura or periosteum (2 cases of dural tension suture, 8 cases of artificial dura, gelatin sponge or dura replacement, and 1 case of intact dura without cutting), except for 1 patient who underwent DC treatment; the details of the operation were not clearly reported. Vega and Hutchins^[11]^ and Gao et al^[9]^ reported cases of bilateral craniectomy. One patient underwent bilateral craniectomy on the day of the injury, and periosteum repair was performed using a gelatin sponge on the left side at the same time. However, repair of the periosteum on the right side was impossible because the skull was severely crushed. Notably, skull regeneration was observed only in the left defect area during follow-up.^[11]^ The other patient underwent dura formation using a dura substitute on both sides, and signs of bone regeneration were subsequently observed on both sides.^[9]^ Taken together, the above information demonstrates the possible role of the periosteum and dura mater in the process of skull regeneration.

In addition, it has also been reported that angiogenesis plays a key role in bone growth and repair in the process of skull regeneration.^[16]^ Some cases cited in this study also suggested that the gentle separation of skin and muscle during the operation to maintain the integrity of blood vessels may be important for skull regeneration.^[12,13]^

### 4.2. Spontaneous skull regeneration and timing of cranioplasty

In this study, skull regeneration was mainly observed in adolescents (17 to 20years old, 50%) with rapid growth and development, and most of the regeneration was observed at 0.5 to 1 years after surgery. In recent literature, cranioplasty has been performed within 3 months of the operation. Early cranioplasty has many advantages, especially in children, such as easier tissue plane dissection, decreased sequelae after craniotomy (trephine syndrome and sinking flap syndrome), and better restoration of symmetrical skull growth.^[17]^ Therefore, cranioplasty should be performed within 3 months after DC to reduce the incidence of complications in young patients, which may partially explain why few young patients with skull regeneration have been reported.

For most patients, postoperative complications (e.g., infection, hydrocephalus, and epilepsy) are major factors that affect the timing of cranioplasty. It has been demonstrated that the cranioplasty performed at 15 to 90 days after craniectomy has the least complications, while cranioplasty performed more than 90 days may reduce the risk of hydrocephalus but increase the risk of epileptic seizure.^[18]^ However, given the clinical existence of skull regeneration, which predominantly occurs in patients with rapid growth and development, an appropriate postponement of the cranioplasty time with close monitoring of postoperative complications and skull regeneration is recommended for young patients with skull defects, aiming to observe bone regeneration and reduce the risk of reoperation. In addition, the regenerated skull is generally thinner than the normal skull; therefore, attention should be paid to the potential risks of craniocerebral injuries in daily life.

## Author contributions

**Conceptualization:** Zilong Liu.

**Data curation:** Huanhuan Yang.

**Investigation:** Huanhuan Yang, Man Liang.

**Methodology:** Huanhuan Yang, Zilong Liu.

**Project administration:** Lijian Su.

**Resources:** Lijian Su.

**Supervision:** Man Liang.

**Writing** - **original draft:** Huanhuan Yang.

**Writing** - **review & editing:** Zilong Liu, Man Liang.
